# Stability of organic carbon pools and sequestration potential as affected under different agroforestry systems

**DOI:** 10.1007/s44297-023-00016-7

**Published:** 2023-11-29

**Authors:** Shaon Kumar Das

**Affiliations:** ICAR Research Complex for NEH Region, Sikkim Centre, Gangtok, Sikkim 737102 India

**Keywords:** Agroforestry, Carbon sequestration, *Alnus nepalensis*, *Schima wallichii*, Carbon pool

## Abstract

There are few data on the possibility of soil organic carbon sequestration for agroforestry systems (AFSs). Rarely are the effects of AFSs established for the regeneration of carbon in degraded soils of the Indian North Eastern Himalayas (susceptible to soil erosion, carbon and nutrient loss) examined. The effects of five different AFSs on the stability of organic carbon pools and sequestration potential were evaluated at the ICAR-Sikkim Centre. Black gram + mandarin + *Alnus nepalensis* had the lowest bulk density at all depths. The highest oxidizable carbon was observed in the black gram + mandarin + *Alnus nepalensis* system, followed by soybean + *Ficus hookerii* + guava, maize + *Schima wallichii*, and soybean + *Ficus hookerii* + guava, followed by Napier. Regardless of soil depth, the buckwheat + mandarin system had a noticeably lower SOC than the other systems. The total soil organic carbon gradually decreased with increasing depth of the soil profile. According to the results, there was little difference in the total active carbon pool in the 0–90 cm depth among the various systems; however, when compared with Buckwheat + Mandarin, it varied significantly (*P* ≤ *0.05*). The mean passive carbon pool in soils ranged from 22.4 to 25.1 Mg ha^−1^ across the land use in the 0–90 cm depth. The least soil microbial biomass carbon (MBC) was observed during the winter season in all systems at different soil depths. The maximum MBC was recorded at 0 to 15 cm depth (476.2 – 302.5 µg dry soil^−1^). By simultaneously cultivating kinds of trees with several uses and agri-horticultural crops, a large-scale adoption of AFSs may replace carbon lost via the development of the crop in degraded soils and offer a viable choice for livelihood.

## Introduction

The comprehensive production management technique known as agroforestry works to maintain and improve the health of agro-ecosystems, encompassing biological cycles, biodiversity, and soil biological activity [1]. Farmers' livelihoods could be improved by the agroforestry system (AFS), which has a significant impact on carbon sequestration, provides a variety of options and chances to increase productivity and incomes, and lessens vulnerability to climate change. [2]. It is considered one of the best options to stabilize the carbon concentrations in the atmosphere to increase the carbon sink [3]. It is widely promoted as a sustainable enhancing practice that combines the best attributes of agriculture and forestry. Agroforestry systems are among the largest terrestrial carbon pools that are the most susceptible to land use and land cover change [4]. Hence, it is indispensable to accomplish AFSs to augment soil organic carbon sequestration and pools [5]. In addition to producing food, fuel, and timber for energy and buildings, as well as providing fodder for cattle, trees on farms also preserve soil by reducing erosion and raising fertility levels. Plantations or croplands downstream benefit greatly from organic nutrients, such as nitrogen and phosphorus, that are supplied by agroforestry-based systems such as Himalayan alder (Alnus nepalensis) [6]. It provides chances for land management techniques through the ecological interactions between adaptable trees and shrubs and crops [7]. Because agroforestry systems often have very high carbon source and sink capacities, there are chances to restore soil fertility and SOC stock [8]. The practice of using Alnus for nitrogen fixation as shade trees has been adopted by indigenous communities to maintain soil fertility and increase productivity [9]. Climate change and carbon dioxide emissions can be impacted by even slight adjustments to the size of this sink. Agriculture is a major contributor to greenhouse gases and plays a significant role in temperature regulation of the earth’s atmosphere; moreover, it also enriches the existing terrestrial carbon pool in the form of soil organic matter [10]. Perennial trees can restore large quantities of carbon in the form of woody masses and dried leaves. Tree-based cropping systems help increase the soil organic carbon pool [11]. Some recent agroforestry systems, such as tree-based crops, large cardamoms based on Alnus, fallow with short-duration/annual crops, and annual fallow relay cropping, have shown promising results in way to sustainable farming systems and help in carbon neutralizing systems to enhance food production. This holistic approach towards sustainable farming will help to combat the problem of carbon emissions and revitalize the goals of productivity and a healthier future [12]. Numerous investigations have revealed that as much as 50–60% of the carbon stock is present below 30 cm of soil depth. In most of the soils in central and eastern Europe, the top 30 cm have been reported to contain approximately 44% of the total C pool found in soil depths of 1 m. [13]. Studies have been conducted on the stability of organic carbon pools and sequestration potential as affected under different agroforestry systems from 0 to 1 m depth. A few studies have also evaluated SOC stocks up to 1.5 m depth, and even fewer have gone as deep as 2 m. However, there is very little information available on the long-term effects of AFSs on the stability of organic carbon pools and sequestration potential. The present experiment was carried out to determine the organic carbon pools and sequestration potential under different agroforestry systems in Sikkim (Northeastern part of the Indian Himalayas) with the following 5 treatment details: Maize + *Schima wallichii*, Buckwheat + Mandarin, Black gram + Mandarin + *Alnus nepalensis*, Soybean + *Ficus hookerii* + guava and Napier at different soil depths (0–90 cm).

## Materials and methods

### Location of study

The work was carried out in an agroforestry block established in the experimental research area of ICAR RC for the NEH Region, Sikkim Center, Gangtok, Sikkim, India. The experimental site was located in the NE-Himalayan region, which is one of the biodiversity hotspots of India situated at 27°32′ N latitude and 88°60′ E longitude with an altitude of 1350 m above mean sea level (MSL). The three distinct monsoonal seasons of summer (March to May), rainy season (June to September), and winter (October to February) are associated with the moderate climate of the experimental site. The pre-monsoon rainfall in Sikkim begins from February–March and reaches its peaks during July with an extreme monthly average of 650 mm. The texture of the experimental location is sandy loam, and hardpan is absent. The soil is slightly acidic (pH 5.5–6.0) in nature. The cultivation or farming method followed in the region is totally organic.

### Experimental details and AFSs management

Five different agroforestry systems, namely, Agrisilviculture, Agrihorticulture, Agri-horti-silviculture, Agrisilvihorticulture, and Grassland systems, were used in this experiment (Table 1). In agrisilviculture, the major vegetation Chilaune (*Schima wallichii*) was intercropped with maize (*Zea mays*), and the age of the Chilaune was 35 years with an altitude of 1225 m MSL. A 12-year-old Mandarin (*Citrus reticulata*) was intercropped with buckwheat (*Fagopyrum esculentum*) in an agrohorticulture system with a nutrient dose per tree of 10–20 kg FYM along with 2–2.5 kg vermicompost and a per hectare dose FYM or mixed compost at 5 tons/ha along with vermicompost at 1.5 t/ha with an altitude of 1175 m MSL. In the agrihortisilviculture system, 12-year-old Mandarin (*Citrus reticulata*) and 35-year-old Uttis (*Alnus nepalensis*) trees were intercropped with black gram (*Vigna mungo*) at an altitude of 1200 m MSL of nutrient dose, as in the previous system. A 20-year-old Fig (*Ficus hookerii*) and a 10-year-old Guava (*Psidium guajava*) tree were intercropped with soybean (*Glycine max*) with nutrient application per hectare FYM at 5–10 t/ha neem cake at 1.0 t/ha + mixed compost at 2.5 t/ha + dolomite at 1.0 t/ha found at an altitude of 1165 m MSL. In the grassland system, a 9-year-old Napier vegetation was found with an altitude of 1085 m MSL. Soil samples were collected from five AFSs, viz., Maize + *Schima wallichii,* Buckwheat + Mandarin, Black gram + Mandarin + *Alnus nepalensis,* Soybean + *Ficus hookerii* + guava, and Napier, which were tested in an RBD design during 2013–2022 (ten years) and replicated three times.

**Table 1:** A brief description of the land use system during past 35 years in the state of Sikkim in Eastern Himalayan Region

Land use system (LUS)	LUS Notation	Land use description		Integrated Nutrient management	Age (year)	Altitude (m msl)
		Tree based/Major vegetation	Intercrop			
Agrisilviculture system	AS	Chilaune (*Schima wallichii*)	Maize (*Zea mays*)	Nil	35	1225
Agrihorticulture system	AH	Mandarin (*Citrus reticulata*)	Buckwheat (*Fagopyrum esculentum*)	Per tree: 10–20 kg FYM along with 2–2.5 kg vermicompost Per hectare: FYM or mixed compost at 5 tons/ha along with vermicompost at 1.5 t/ha	12	1175
Agrihortisilviculture system	AHS	Mandarin (*Citrus reticulata*) Uttis (*Alnus nepalensis*)	Black gram (*Vigna mungo*)	Per tree: 10–20 kg FYM along with 2–2.5 kg vermicompost Per hectare: FYM or mixed compost at 5 tons/ha	12 35	1200
Agrisilvihorticulture system	ASH	Fig (*Ficus hookerii*) Guava (*Psidium guajava*)	Soybean (*Glycine max*)	Per tree: 10–20 kg FYM along with 2–2.5 kg vermicompost Per hectare: FYM at 5–10 t/ha neem cake at 1 t/ha + mixed compost at 2.5 t/ha + dolomite at 1 t/ha	20 10	1165
Grassland	GS	Napier	Nil	Nil	9	1085

### Soil collection and sample preparation

Soil samples from well-established agroforestry systems intercropped with different crops, such as maize, buckwheat, black gram, and soybean, were collected from 20 different sites (for each land use, 4 sites were selected) at depths of 0–15 cm, 15–30 cm, 30–45 cm, 45–60 cm, 60–75 cm, and 75–90 cm for this study. To create a composite soil sample from each depth, four randomly selected spots from each AFS were sampled and blended. A 250 g bulk soil sample was taken at the appropriate depths to evaluate SOC and other soil characteristics. Prior to and following drying, the soil samples and containers were weighed in the laboratory using an electronic scale. The bulk soil samples collected were air-dried at 40 °C, soil clods were broken manually, and extraneous roots and other debris were removed. The soil was ground gently and sieved using a 2 mm mesh. Samples of sieved soil were placed in airtight plastic bags to await examination of the physiochemical characteristics of the soil. A portion of each composite fresh soil sample from each plot was kept frozen so that dehydrogenase activity and soil microbial biomass carbon (SMBC) could be measured. The wet oxidation method was used to analyse the soil organic carbon (SOC) of the samples.

### Total organic carbon estimation

Organic carbon can be detected and oxidised in two stages using TOC analyzers (Elementar; Vario TOC choose model). In both liquid and solid samples, it is also capable of detecting total carbon (TC) and total inorganic carbon (TIC). The sample was fed into the combustion furnace in this apparatus. After that, it is heated using a platinum catalyst to a high temperature of 680 °C to undergo combustion. Decomposition takes place here and is transformed to CO2. The produced CO2 was cooled, the humidity was removed, and an NDIR (Non Dispersive Infrared) detector was used to detect it.

### Oxidizable organic carbon estimation

Jackson (1967) described the Walkley–Black chromic acid wet oxidation method, which provides the basis for determining the amount of oxidizable organic carbon. In a 500 ml flask, 1 g of soil was added, followed by 10 ml of 1 N K_2_Cr_2_O_7_. Next, 20 ml of concentrated H_2_SO_4_ was poured straight into the stream into the suspension after gently swirling the flask to distribute the dirt in the solution. Immediately swirled the flask gently and then more vigorously for 1.0 min. After the flask had been left to stand for approximately thirty minutes, 200 mL of water was added, and it was once more allowed to cool. Subsequently, the solution was shaken well and titrated against ferrous ammonium sulfate (FAS) after 10 mL of orthophosphoric acid and 1 mL of diphenylamine indicator were added. Green replaced the previous blue‒violet hue. To standardize FAS, a blank determination was also made in the same way without dirt.

$$\mathrm{Organic}\;\mathrm{carbon}\;(\%)=\lbrack10\;(\mathrm B-\mathrm T)/\mathrm B\rbrack\times\lbrack(0.003\times100)/\mathrm{wt}\;\mathrm{of}\;\mathrm{soil}\;(\mathrm{gm})\rbrack$$

where B = Amount of FAS solution utilised to titrate a blank

T = Amount of FAS solution used to titrate a sample of soil.

### Active and passive carbon pools

The very labile pool (C frac_1_) and labile pool (C frac_2_) together designated the active pool of organic carbon. This was because of their very easy oxidisability using 12 and 18 N H_2_SO_4._ Passive pools are those that are jointly designated as C_tot_ less labile (C frac_3_) and nonlabile (C frac_4_) pools.

### Statistical analysis

To compare the treatment averages at *p* = 0.05, the least significant difference (LSD) test was employed. At *p* = 0.05, LSD was used to compare each AFS with croplands and each AFS with other AFSs. The treatment means that are shown in the results tables and annotated with different letters are substantially different from one another by LSD at *p* = 0.05.

## Results and discussion

### Effect of land use management on the pH and bulk density of soil

**Fig. 1:**
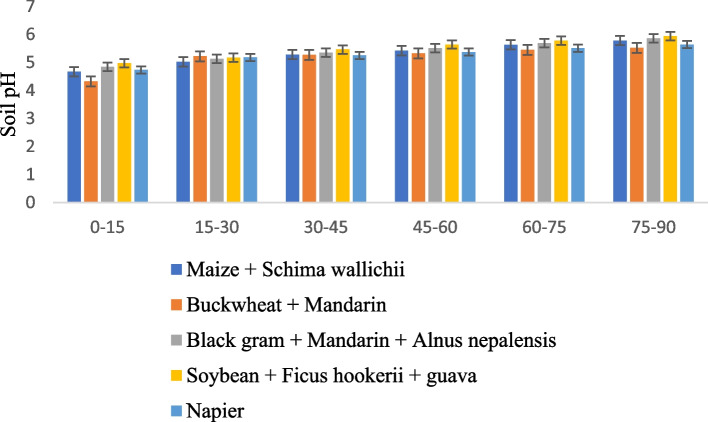
Effect of different agroforestry system on soil pH

**Fig. 2:**
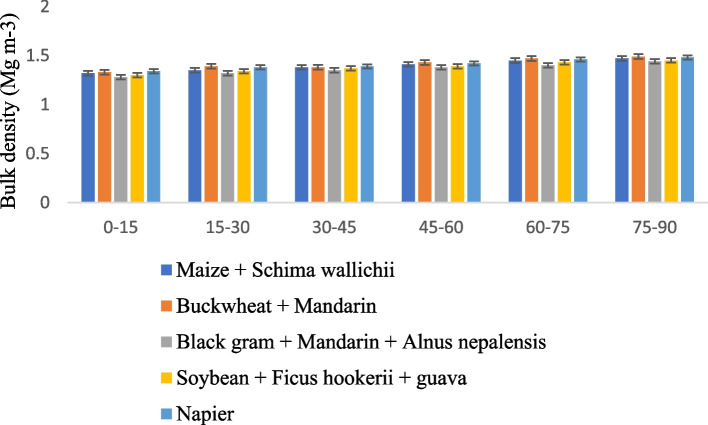
Effect of different agroforestry system on soil bulk density (Mg m.^−3^)

Soil bulk density under different AFSs varied significantly. The effect of land use management on soil pH and bulk density increased with an increase in soil depth down to 90 cm in all agroforestry systems, as presented in Figs. 1 and 2. Across the various land use systems, the lowest pH and pb values were found at 0–15 cm depth, and the highest values were found between 75 and 90 cm depth. The mean soil pH ranged from 5.18 to 5.49 in different land use systems. The highest mean pH value was recorded in the Soybean + *Ficus hookerii* + guava-based cropping system, while Buckwheat + Mandarin observed the lowest pH value of 5.18. It is found that land use systems across the soil at depths of 0–15 cm are acidic, and depths below the topsoil are less acidic in nature. At all soil depths, land use patterns had a substantial impact on the pH of the soil. Across soil depths, land use management techniques significantly impacted bulk density. Black gram + Mandarin + *Alnus nepalensis* had the lowest bulk density at all depths. The highest bulk density was observed in Buckwheat + Mandarinthe land use system. The bulk density of soils under various AFSs was lower than that of the buckwheat + mandarin described here, contrary to the findings of many other researchers' studies. It is a proven truth that cultivating various tree species with livestock, horticulture, or fodder crops increases the biomass of the litter [14–17]. A system like this also enriches the soil with organic matter, increases soil porosity due to deeper and more numerous roots, and builds up a large amount of OM because it produces a large amount of aboveground biomass and detritus materials [18]. As a result, a lower bulk density than that of the buckwheat + mandarin system was observed under various AFSs. Although the degree of changes in bulk density varied across systems, these could be explained by differences in the root architecture and fine root biomass among the different species of trees, which have an impact on the root volume and rooting pattern. [19]. Under various AFSs, the depth-wise fluctuation in soil bulk density was also noted. As bulk density is a good indicator of soil quality, the impact of AFSs on soil quality might be seen in the lower soil bulk density under tree-based systems compared to those under arable lands [20].

### Land use system on oxidizable and soil organic carbon

**Fig. 3:**
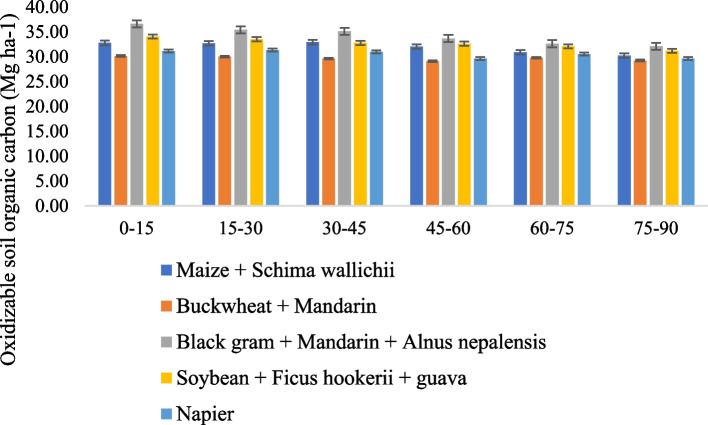
Effect of different agroforestry system on oxidizable soil organic carbon

**Fig. 4:**
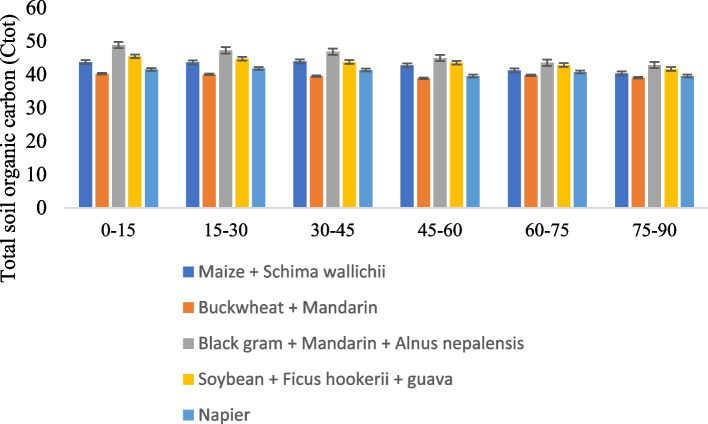
Effect of different agroforestry system on total soil organic carbon

The impact of land use management on the total and oxidizable carbon in soil is presented in Figs. 3 and 4. The oxidizable soil organic carbon varied from 29.65 to 34.25 Mg C ha^−1^ soil across the land use system. The highest oxidizable carbon was observed in Black gram + Mandarin + *Alnus nepalensis* and the use system, followed by Soybean + *Ficus hookerii* + guava, Maize + *Schima wallichii*, and Soybean + *Ficus hookerii* + guava, followed by Napier. The C_oc_ content within the land use system was determined to be non-significant. However, while compared with Buckwheat + Mandarin, it varied significantly (*P* ≤ 0.05). SOC is significantly impacted by land use management at all soil depths. Between 39.5 and 45.7 Mg ha^−1^ was the mean SOC for each of the land use systems. Black gram + Mandarin + *Alnus nepalensis* had a much greater SOC at all depths, followed by Soybean + *Ficus hookerii* + guava, based on cropping compared to those under alternative land use schemes. The total soil organic carbon gradually decreased with increasing soil column depth. Buckwheat + Mandarin, regardless of soil depth and land use, had a far lower SOC than other land use regimes [21]. Multiple studies have suggested that the growth of trees in agroforestry systems alongside horticultural and arable crops boosted carbon pools and sequestration. Nevertheless, Upson and Burgess (2013) findings that indicated a loss of carbon in the soil beneath trees largely contradict the SOC conclusion in this study [22]. Additionally, the rise in carbon pools in different AFSs may be brought on by an augmentation in yearly C-enriched inputs from biomass brought on by litterfall followed by gradual breakdown of the root system. Additionally, a significant amount of soil aggregates develop, carbon-rich micro-aggregates being encased in macro-aggregates, the defence of soil aggregates against the direct action of precipitation, the formation of organo-mineral compounds, the control of soil temperature by lowering solar radiation insolation, and other factors, individually or collectively, to raise the SOC content [23]. Additionally, the current study location is located in a region with abundant rainfall, which encourages the rapid growth of plant shoots and roots and thus results in more carbon pools under AFSs than croplands [24–26].

### Land use system on the active pool and passive pool

**Fig. 5:**
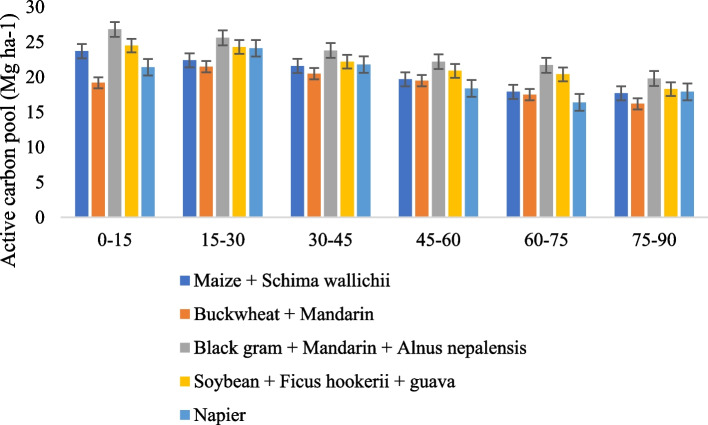
Effect of different agroforestry system on active carbon pools in soil

**Fig. 6:**
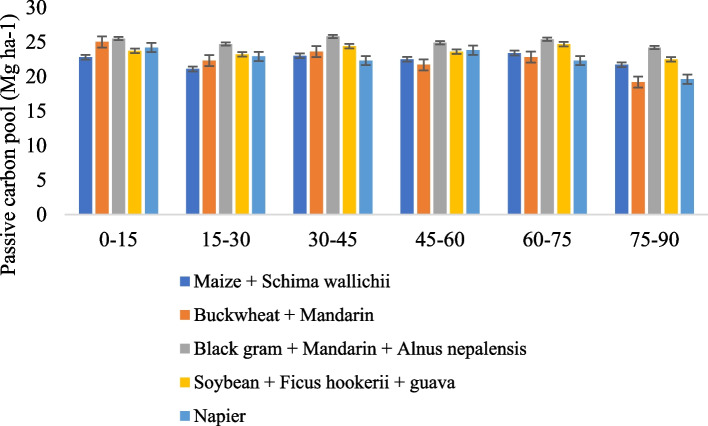
Effect of different agroforestry system on passive carbon pools in soil

**Fig. 7:**
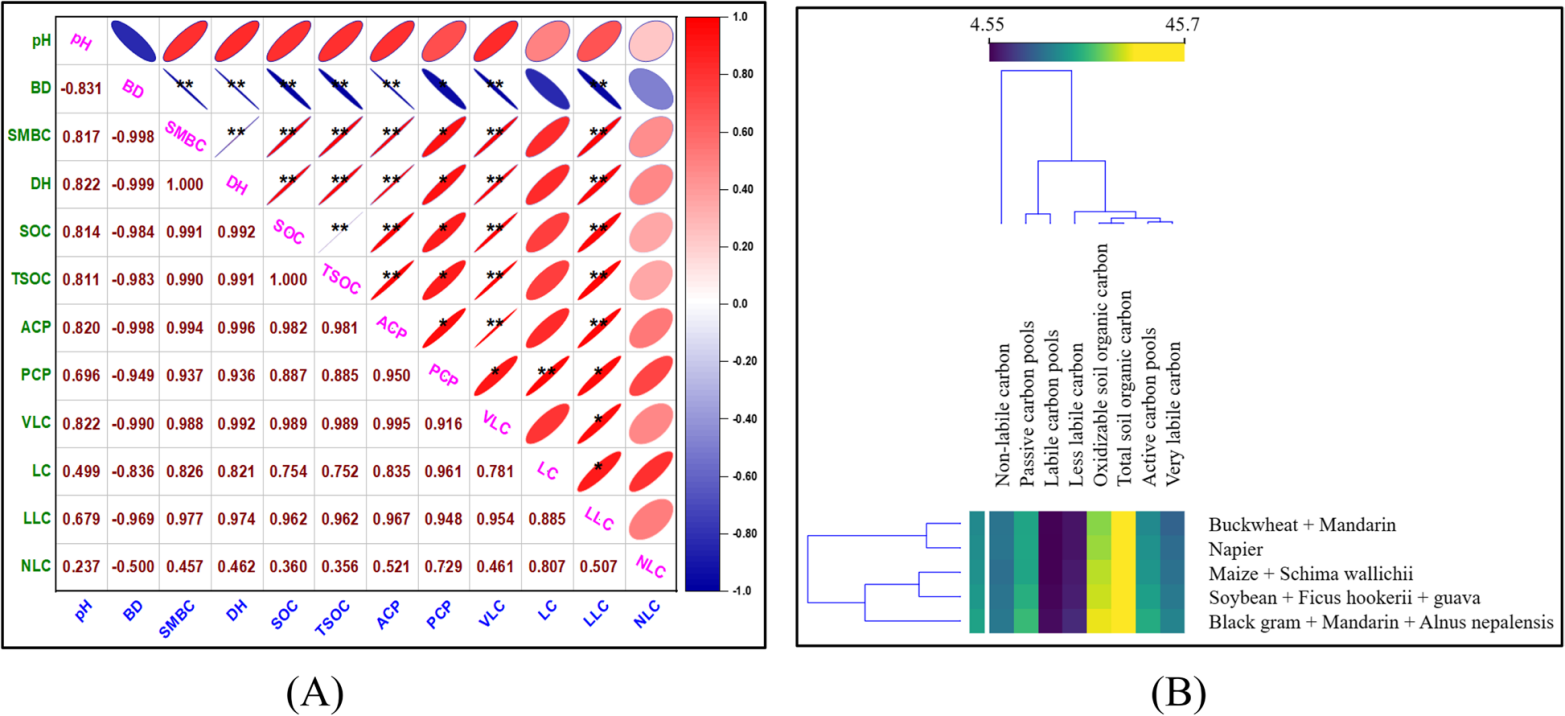
**A** Correlogram of Pearson correlation coefficients (r) matrix between different carbon fractions. **B** A clustering heat map depicting various carbon fractions as affected by biochar

Across all soil depths, the land use shift had a considerable impact on both the active carbon (AC) and passive carbon (PC) pools (Figs. 5 and 6). There is a relatively labile pool of oxidizable organic carbon that correlates to the active carbon pool. The mean AC pool in soils ranged from 19.1 to 23.3 Mg ha-1 across the land use in the 0–90 cm depth. Among the land use cropping systems, the Black gram + Mandarin + *Alnus nepalensis* and Soybean + *Ficus hookerii* + guava substantially (*P* ≤ 0.05) increased the active carbon pool over Buckwheat + Mandarin in 0-15 cm at the 0–15 cm depth. Regardless of the land use schemes, the largest documented maximum active carbon pool was found in the 0–15 cm depth in Black gram + Mandarin + *Alnus nepalensis* and lowest in Buckwheat + Mandarin (19.2 Mg ha^−1^). According to the findings, the whole reservoir of active carbon in the 0–90 cm depth among the different land use systems showed no discernible change, but while contrasted with Buckwheat + Mandarin, it varied significantly (*P* ≤ 0.05). The highest amount of total active carbon pool was noted under Black gram + Mandarin + *Alnus nepalensis* (23.3 Mg ha-1) followed by Soybean + *Ficus hookerii* + guava (21.8 Mg ha-1). It was shown that the minimum total active carbon pool in Buckwheat + Mandarin (19.1 Mg ha-1) land use practices. The less labile and non-labile pool of oxidizable organic carbon is equivalent to the passive carbon pool. The findings verified that among soils from various land use systems, there was little change in the total passive carbon pool at a depth of 0–90 cm. However, within the 0–15 cm depth, the passive C pool's statistically identical value was observed between the Black gram + Mandarin + *Alnus nepalensis* and Buckwheat + Mandarin land use systems. However, at the 30–45 cm depth, Black gram + Mandarin + *Alnus nepalensis* system had a noticeably greater quantity of passive carbon pools (25.8 Mg ha^−1^) followed by Soybean + *Ficus hookerii* + guava (24.4 Mg ha^−1^) comparison of land usage with other land uses. On the other hand, soils under Maize + *Schima wallichii* had the lowest PC pool (22.8 Mg ha^−1^) at 0–15 cm and 15–30 cm (21.1 Mg ha^−1^) depths. However, at the 30–45 cm soil depth, Napier land use had the lowest PC pool (22.3 Mg ha^−1^). Buckwheat + Mandarin land use system recorded the lowest PC pool (21.7 Mg ha^−1^) at 45–60 cm and at 75–90 cm (19.2 Mg ha^−1^) depth. The mean PC pool in soils ranged from 22.4 to 25.1 Mg ha^−1^ across the land use in the 0–90 cm depth. The Correlogram of the Pearson correlation coefficient (r) matrix between different carbon fractions and a clustering heatmap depicting various carbon fractions as affected by biochar are presented in Fig. 7. The percentage of each type of carbon fraction in soils varied as a result of the cultivation of tree species alongside agricultural and horticultural crops. Regardless of land-use patterns, generally speaking, pools of the less labile part of carbon became deeper. Regardless of the land use system, the increased proportion of highly labile and labile carbon in the top 0–30 cm of soil may be attributable to the regular supply of fresh carbon in the shape of branches and foliage [27]. More non-labile C is found in soils that are deeper than 30 cm. The larger percentage of less labile carbon and non-labile carbon beneath AFSs may be attributable to chemical defense, conversion to char C, depth defense, limited microbial activities, and the development of organic mineral complexes. In the current investigation, variation in the C percentage in the AFSs was also noted. Such an investigation found that the C pools in soils with various AFSs had an erratic trend. This discrepancy might be a trait of the species or it might be caused by other factors that require more research [28].

### Land use system on soil microbial biomass carbon and soil dehydrogenase activities

The data shown in Table 2 of soil microbial biomass carbon (MBC) revealed a regressive decline as depth increased across the land use systems. In all land uses and at all soil depths, the rainy season produced the greatest soil MBC values. Wintertime had the lowest soil MBC across all land uses with varying soil profiles. Between 0 and 15 cm of depth, the greatest soil MBC was measured (476.2 – 302.5 µg dry soil^−1^). Significantly higher concentrations of MCB were observed in soil under Black gram + Mandarin + *Alnus nepalensis*, Soybean + *Ficus hookerii* + guava and Maize + *Schima wallichii* land use systems than in soils beneath Napier and Buckwheat + Mandarin land use systems across all depths and seasons. The data presented in Table 3 show the impact of soil dehydrogenase management on land use activities across the season, indicating that in all land use regimes, the wet season had the highest DHA activity in the soils, whereas the winter months had the lowest DHA activity at all soil depths [29]. As the soil profile depth increased, the DHA activity decreased. Maximum DHA movement was monitored in 0–15 cm soil and minimum in 75–90 cm soil in all AFSs throughout all periods. Among the different land use systems, the Black Gram + Mandarin + *Alnus nepalensis*, Soybean + *Ficus hookerii* + guava and Maize + *Schima wallichii* land use systems exhibited noticeably greater DHA activity in the depths during every season than the Napier and Buckwheat + Mandarin land use systems. Moreover, DHA activity in the soils under the Napier and Buckwheat + Mandarin systems was not appreciably different at 30–45 cm and 60–75 cm depths during the winter and summer periods but differed significantly at rest of the depths during rainy seasons [30]. The breakdown of organic materials in soil is mediated by MBC and DHA. MBC and DHA are delicate markers of how land is used and managed practices because they both react more quickly to changes in SOM quality [31]. With increasing soil depth across all AFSs, the DHA and MBC concentration activity decreased. A lower SOC amount and microbial population as a result of increased sub-soil compaction and less OM deposition may be the cause of this trend. In the current study, significant effects of land use on MBC and DHA activities were found. The intake of a variety of amounts of organic wastes and biomass is responsible for the greater amount of MBC in forest systems [32–34]. All land uses in the current study showed seasonal differences in the activity of MBC and DHA, which could be explained by changes in soil temperature as well as temperature throughout the year. An ideal microbial population and DHA activity depend on the soil having a sufficient moisture level [35]. During the rainy season, ideal temperature, sufficient soil moisture, and a decent supply of substrate carbon could have catalysed microbial activity, while low temperatures and decreased soil moisture during the winter months slowed the activities of soil microorganisms and enzymes [36].

**Table 2:** Effect of different agroforestry system on soil microbial biomass carbon (µ g dry soil^−1^) across the seasons

Land uses	Soil depth (cm)						
	Summer season						
	0–15	15–30	30–45	45–60	60–75	75–90	Mean
Maize + *Schima wallichii*	343.2^c^	267.8^b^	218.7^b^	152.8^c^	125.4^c^	104.6^b^	202.1^c^
Buckwheat + Mandarin	321.5^e^	244.6^d^	184.8^d^	126.4^e^	108.5^d^	95.5^c^	180.2^d^
Black gram + Mandarin + *Alnus nepalensis*	374.8^a^	298.5^a^	266.5^a^	192.7^a^	164.6^a^	139.8^a^	239.5^a^
Soybean + *Ficus hookerii* + guava	358.6^b^	272.5^b^	248.8^b^	175.3^b^	142.7^b^	115.6^b^	218.9^b^
Napier	330.7^d^	250.7^c^	192.3^c^	138.2^d^	116.2^d^	98.7^c^	187.8^d^
Land uses	Soil depth (cm)						
	Rainy season						
	0–15	15–30	30–45	45–60	60–75	75–90	Mean
Maize + *Schima wallichii*	447.5^b^	348.7^c^	270.5^b^	246.7^c^	207.8^b^	176.2^b^	282.9^b^
Buckwheat + Mandarin	421.2^c^	318.6^d^	237.5^d^	201.5^d^	175.6^c^	136.7^d^	248.5^c^
Black gram + Mandarin + *Alnus nepalensis*	476.2^a^	382.5^a^	324.2^a^	284.8^a^	247.2^a^	222.8^a^	323.0^a^
Soybean + *Ficus hookerii* + guava	461.7^a^	367.2^b^	305.8a	268.5^b^	226.7^b^	208.5^a^	306.4^a^
Napier	425.8^c^	332.8^c^	255.7^c^	222.2^d^	182.5^c^	158.5^c^	262.9^c^
Land uses	Soil depth (cm)						
	Winter season						
	0–15	15–30	30–45	45–60	60–75	75–90	Mean
Maize + *Schima wallichii*	316.7^b^	292.2^b^	234.7^c^	206.7^b^	145.8^c^	96.2^b^	215.4^b^
Buckwheat + Mandarin	286.2^d^	273.4^c^	218.0^d^	186.5^d^	116.7^d^	76.7^c^	192.9^d^
Black gram + Mandarin + *Alnus nepalensis*	335.4^a^	321.6^a^	256.3^a^	222.5^a^	174.8^a^	115.8^a^	237.7^a^
Soybean + *Ficus hookerii* + guava	322.8^a^	308.5^a^	243.5^b^	215.8^b^	152.5^b^	108.7^a^	225.3^b^
Napier	302.5^c^	281.7^b^	222.2^d^	194.2^c^	128.2^d^	84.5^c^	202.2^c^

^**^Means followed by different letters (a-e) are significantly different with respect to the LSD (least significant difference) values at *p* = 0.05. Significant differences shown are with regard to land uses

**Table 3:** Effect of different agroforestry system on soil dehydrogenase activities (µ g TPF-1 soil h^−1^) across the seasons

Land uses	Soil depth (cm)						
	Summer season						
	0–15	15–30	30–45	45–60	60–75	75–90	Mean
Maize + *Schima wallichii*	14.45^c^	11.52^c^	10.13^c^	8.46^c^	7.23^c^	6.75^c^	9.8^c^
Buckwheat + Mandarin	12.26^d^	9.75^d^	9.26^d^	6.58^e^	6.12^d^	4.52^e^	8.1^d^
Black gram + Mandarin + *Alnus nepalensis*	17.36^a^	14.46^a^	12.65^a^	10.54^a^	9.22^a^	8.45^a^	12.1^a^
Soybean + *Ficus hookerii* + guava	16.28^b^	13.35^b^	11.35^b^	9.25^b^	8.56^b^	7.21^b^	11.0^b^
Napier	12.52^d^	9.86^d^	9.74^d^	7.84^d^	6.52^d^	5.35^d^	8.6^d^
Land uses	Soil depth (cm)						
	Rainy season						
	0–15	15–30	30–45	45–60	60–75	75–90	Mean
Maize + *Schima wallichii*	23.46^c^	16.68^c^	12.46^c^	9.78^c^	9.23^c^	7.84^c^	13.2^b^
Buckwheat + Mandarin	19.25^d^	14.36^e^	10.25^e^	7.56^e^	7.32^e^	6.24^d^	10.8^d^
Black gram + Mandarin + *Alnus nepalensis*	26.42^a^	21.26^a^	16.76^a^	14.68^a^	11.75^a^	9.46^a^	16.7^a^
Soybean + *Ficus hookerii* + guava	24.68^b^	18.72^b^	13.54^b^	12.35^b^	10.52^b^	8.26^b^	14.7^b^
Napier	20.62^d^	15.15^d^	11.32^d^	8.64^d^	8.54^d^	6.88^d^	11.9^c^
Land uses	Soil depth (cm)						
	Winter season						
	0–15	15–30	30–45	45–60	60–75	75–90	Mean
Maize + *Schima wallichii*	13.36^c^	11.12^c^	9.24^c^	9.35^b^	8.95^b^	7.21^b^	9.9^c^
Buckwheat + Mandarin	12.08^d^	9.88^d^	8.15^d^	8.02^c^	7.89^c^	6.44^c^	8.7^d^
Black gram + Mandarin + *Alnus nepalensis*	16.75^a^	13.46^a^	12.14^a^	10.68^a^	9.67^a^	8.72^a^	11.9^a^
Soybean + *Ficus hookerii* + guava	14.56^b^	12.28^b^	11.65^b^	9.75^b^	8.76^b^	7.56^b^	10.8^b^
Napier	12.44^d^	10.54^d^	8.75^d^	8.42^c^	8.28^b^	6.65^c^	9.2^c^

^**^Means followed by different letters (a-e) are significantly different with respect to the LSD (least significant difference) values at *p* = 0.05. Significant differences shown are with regard to land uses

## Conclusion

To stop global climate disasters, it is critical to estimate the carbon store in agroforestry. The current survey found five agroforestry systems to be common in the Sikkim Himalayas study area, namely Agrisilviculture, Agrihorticulture, Agri-horti-silviculture, Agrisilvihorticulture and Grassland. This study provides a helpful method for deciding which agroforestry systems to adopt to maximize carbon stocks efficiently and minimize the effects of global warming in another vulnerable region in the northeastern Indian Himalayas. The study was able to show that, in contrast to crop cultivation in North Eastern Himalayan degraded areas, organic carbon pools and sequestration potential rose under various AFSs. Thus, from the carbon management index perspective, Agrihortisilviculture system has a greater capacity to mitigate carbon emissions, followed by Agrisilvihorticulturethe system and Agrisilviculture system, which, depending on local variables, can be both economically and ecologically feasible. Compared to the carbon found in soils beneath croplands, the study demonstrated that the installation of AFSs on sloping or degraded hills increased a significant amount of carbon and significantly improved the quality of the soil. By simultaneously cultivating multipurpose tree species, agricultural crops, and large-scale adoption of AFSs might replace carbon released during agricultural farming on degraded land and offer a possible means of subsistence. Policymakers and farmers in the Himalayan area, as well as those in neighbouring ecoregions, will benefit from the novel insights produced by this study to create and rework agricultural techniques for sloping, hilly terrain that is prone to soil erosion, carbon loss, and low yield.

## Data Availability

The data presented in this study are contained within the article.
